# Behavioral addictions in euthymic patients with bipolar I disorder: a comparison to controls

**DOI:** 10.1186/2194-7511-1-27

**Published:** 2013-12-23

**Authors:** Ran Sapir, Ada H Zohar, Yuly Bersudsky, RH Belmaker, Yamima Osher

**Affiliations:** Department of Psychology, Ruppin Academic Center, Emek Hefer, Israel; Department of Psychiatry, Faculty of Health Sciences, Ben Gurion University of the Negev, Beer Sheva, Israel

**Keywords:** Bipolar disorder, Behavioral approach system, Behavioral addictions, Temperament

## Abstract

**Background:**

Bipolar disorder may be associated with a hypersensitive behavioral approach system and therefore to increased reward sensitivity. The objective of this study is to explore the interrelationships between bipolar disorder, behavioral addictions, and personality/temperament traits in a group of euthymic outpatients with bipolar I disorder and in a group of comparison subjects.

**Methods:**

Fifty clinically stable patients and 50 comparison subjects matched for age, sex, and educational level were administered the Temperament and Character Inventory-140 and the Behavioral Addiction Scale.

**Results:**

The patient group scored significantly higher than comparison subjects for two benign behavioral addictions (music, shopping) as well as for smoking. Comparison subjects scored higher on two harmful behavioral addictions (drugs, alcohol). Novelty Seeking was positively correlated with harmful addictions, and Cooperativeness was negatively correlated with harmful addictions, in both groups.

**Discussion:**

The hypersensitive behavioral approach system model of bipolar disorder would predict higher levels of various addictions in bipolar patients as compared to controls. In this study, this was true for three behavioral addictions, whereas controls showed higher levels of behavioral addiction to drugs and alcohol. This may be because the patients in this study are stable, have received considerable psychoeducation, and are relatively adherent to their medication recommendations. Temperament and character traits may play roles both as risk and protective factors regarding behavioral addictions.

## Background

It has been suggested (Depue and Iacono 1989; Alloy and Abramson 2010) that bipolar disorder is associated with a hypersensitive behavioral approach system (BAS). According to this model (Gray 1973; Gray and McNaughton 2004), there are three brain systems which affect behavior and emotion. The fight/flight/freeze system (FFFS) organizes behavior in the face of aversive stimuli which can be avoided or escaped. The behavioral inhibition system (BIS) is activated by association with punishment or termination of reward, while the BAS is activated in response to reward or the termination of punishment (Franken et al. 2006). Once activated, the BAS initiates motor activity, positive affect, and motivation for reward; the BAS has been related to the activation of several dopamine pathways and brain regions including the nucleus accumbens, the orbitofrontal cortex, the anterior cingulated cortex, and the dorsolateral prefrontal cortex (Depue and Collins 1999; Berns et al. 2001).

Alloy and Abramson (2010) offered a dysregulation perspective to explain how hypersensitivity in the BAS system is expressed in individuals vulnerable to bipolar spectrum disorders. It would appear that a sensitive BAS, hyper-reactive to cues involving reward or termination of punishment, becomes dysregulated easily. Such sensitivity may lead to great variability in BAS activation over time and across situations in response to stimuli. An overactive BAS could result in manic-like behavior (hyperactivity, enhanced appetitive behavior, euphoric or irritable mood), whereas an underactive BAS could result in depression-like behavior (anhedonia, lack of motivation, passivity) (Urosevic et al. 2008).

A possible role for BAS dysregulation in the bipolar spectrum in general and in patients with bipolar I specifically has been supported in several studies using the self-report BIS/BAS scale (Carver and White 1994). Alloy et al. (2009c) and Meyer et al. (2001) found higher BAS scores in euthymic bipolar I patients as compared to healthy controls. Similar results were reported by Salavert et al. (2007) using a different self-report measure of BAS sensitivity. In two studies comparing bipolar spectrum participants (bipolar II and cyclothymic) to demographically matched controls, the bipolar spectrum group reported higher total scores in the BAS (Alloy et al. 2008; Alloy et al. 2009b). In one prospective high-risk design study, adolescents with high BAS scores, over 12 to 13 years of follow-up, had a higher probability and a shorter time to onset of bipolar spectrum disorders than did a group with moderate scores (Alloy et al. 2012).

Assuming that bipolar disorder (BP) is related to a hypersensitive BAS and therefore to increased reward sensitivity, it would seem logical to predict unusually high rates of substance use, abuse, and addiction among patients with BP disorder. In fact, studies have shown prevalence of substance abuse among patients diagnosed with bipolar I disorder to range between 35% and 60%, approximately three to nine times the prevalence in the general population (Lagerberg et al. 2010; Merikangas et al. 2007; ten Have et al. 2002). Additionally, there is higher lifetime prevalence of substance use disorders (SUDs) in bipolar disorder as compared to other mood disorders (Quello et al. 2005).

Possible specific links between the BAS and SUDs have been explored in non-patient as well as in patient samples. Franken and Muris (2005) found that the BAS dimension of fun seeking correlated positively with a number of illegal substances which undergraduate students had used, frequency of binge drinking, and overall quantity of alcohol consumed; the BAS dimension of drive also correlated (weakly) with the amount of illegal substances used. In patients with bipolar spectrum disorders, Alloy et al. (2009a) found that high BAS sensitivity predicted greater substance use problems at follow-up, even after controlling for lifetime SUDs.

These findings suggest that BAS hypersensitivity is related to both vulnerability to SUDs and to mood symptoms. Interestingly, the sensitivity of the reward system is hypothesized to involve dopaminergic projections (Urosevic et al. 2008) which are also involved in the neurobiology of both substance and behavioral addictions (Grant et al. 2006).

Substance use is one element of the larger phenotype of behavioral addiction. Behavioral addictions (BAs) are similar to substance addiction in exhibiting diminished control, but the main expression of the addiction is its behavioral focus - it does not necessarily include the ingestion of a psychoactive substance. BAs are experienced as pleasurable and give relief and excitement whether the addiction is to music, exercise, gambling, or drugs (Meyer et al. 2007).

Substance abuse and behavioral addictions have much in common, including ‘natural history, phenomenology, tolerance, co-morbidity, overlapping genetic contribution, neurobiological mechanisms, and response to treatment’ (Grant et al. 2010, p. 233). It is not surprising, therefore, that the prevalence of behavioral addictions has been found to be higher in patients with bipolar disorder than among healthy controls - 33% vs. 13% - in one study (Di Nicola et al. 2010).

It is not clear what role personality factors may play in the relationship between BP disorder and BAs. In particular, traits related to disinhibition or behavioral undercontrol may mediate the diathesis to both bipolar disorder and behavioral addictions. The psychobiological model of personality, developed by Cloninger (Cloninger et al. 1993; Cloninger and Svrakic 1997), is a two-tier system. The first tier, the temperament traits, includes Harm Avoidance (HA), Novelty Seeking (NS), Reward Dependence (RD), and Persistence (PS). The second tier, the character traits, includes Self-directedness (SD), Cooperativeness (CO), and Self-transcendence (ST). Mardaga and Hansenne (2007) explored the connection between these two models in an undergraduate sample of 150 participants and found that BAS scores were predicted by high novelty seeking and high persistence scores. We decided to further explore the interrelationships between bipolar disorder, behavioral addictions, and personality/temperament traits by comparing responses on two self-report questionnaires, one for behavioral addictions and one a measure of personality according to Cloninger’s model, administered to euthymic adult bipolar patients and to a matched healthy control group. This study was approved by the BGU Institutional Review Board.

## Methods

The patient group consisted of 50 adult (ages 18 to 65) euthymic patient volunteers diagnosed as having bipolar I disorder as diagnosed by DSM-IV criteria based on chart review and clinical interview and were recruited from the well-defined bipolar patient population of the ambulatory Mood Disorders Clinic of the Beer Sheva Mental Health Center. Every patient recruited was well known to the clinic staff, and some have been treated by the same team for over 20 years. (Osher et al. 2010). Exclusion criteria were dementia or language skills inadequate for comprehension of the questionnaires in available languages. The control group was recruited from the community and consisted of 50 healthy volunteers matched with patients for age (±5 years), sex, gender, and educational level. The participants aged 40 and older were recruited in the Sharon area in Israel and were drawn from an ongoing longitudinal study of personality (Cloninger and Zohar 2011). The younger participants were recruited using a snowball technique.

### Procedure

Eligible patients coming to the outpatient mood disorders clinic for their routine appointments were invited to participate in the study after euthymic status had been confirmed by consensus between the two treating clinicians (YO and RHB or YB). The goals of the study were explained, and signed informed consent documents were obtained. The two self-report measures were administered in the patient’s preferred language, either by paper and pencil format or directly into a notebook computer, according to the preference of the participant. Healthy control participants were contacted by the experimenter (RS) and invited to take part in this study. In all cases, the experimenter (RS) was present to address any questions or concerns as they arose.

### Instruments

All participants were administered the 140-item version of the Temperament and Character Inventory-Revised (TCI-R), a Likert-scale self-report questionnaire (Cloninger 1999; Hebrew: Zohar and Cloninger 2011), measuring four temperament dimensions (Novelty Seeking, Harm Avoidance, Reward Dependence, and Persistence) and three character dimensions (Self-directedness, Cooperativeness, and Self-transcendence).

Addictive tendencies were measured by the Behavioral Addiction Scale, a 96-item questionnaire developed by Meyer et al. (2007). Participants were asked eight addiction-related questions for each of the 12 domains: alcohol, cigarettes, drugs, caffeine, chocolate, exercise, gambling, music, internet, shopping, work, and love/relationships. Based on addiction-related criteria developed by Brown (1993), all participants were asked (1) whether they regarded the substance/activity as important (salience), (2) whether they regarded it as enjoyable (euphoria), (3) whether they felt the need to consume more/engage in it more to achieve the same effect (tolerance), (4) whether they felt discomfort upon discontinuation (withdrawal), (5) whether the substance/activity had affected their relationships with others (conflict 1), (6) whether the substance/activity had affected other life domains, such as work or hobbies (conflict 2), (7) whether they had unsuccessfully tried to quit (relapse and reinstatement), and (8) whether they regarded themselves as addicted to the substance/activity (identification with addiction). Five-point response scales ranging from ‘very false for me’ (1) to ‘very true for me’ (5) were used for each item. Thus, a total of 96 addiction-related items were administered. The behavioral addictions were divided to two categories: harmful addictions, including alcohol, drugs, cigarettes, and gambling; and benign addictions, including chocolate, caffeine, exercise, shopping, internet, love relationships, music, and work. Separate scores were computed for harmful and benign behavioral addictions. Total behavioral addiction scores were computed as the sum of both types of BAs.

## Results

There were no significant differences between the patient and control groups with respect to age (42.2 ± 11.92 vs. 43.2 ± 16.23; *t* = 0.18, *p* = 0.85) or sex (27 females, 23 males in each group). Control participants were slightly more educated than patients on average (15.1 ± 2.85 vs. 13.6 ± 2.36; *t* = −2.94, *p* = 0.005).

Bipolar patients had an average age of onset of 23.6 years (SD = 7.3, range 12 to 47) and had been ill for an average of 19.3 years (SD = 10.8, range 1 to 47). All patients were receiving psychotropic medication, with 35 on lithium, 14 on anticonvulsants, 26 on atypical antipsychotics, and another 6 on typical antipsychotics; five patients received an antidepressant. The total is more than 50 patients as only 18 patients were on monotherapy, while 28 patients received two medications and 4 patients received three or more.

### TCI

Repeated measures ANOVA with summed scale scores as the within-subject factors showed no significant effect for group [*F* (1,98) = 2.8, *p* = 0.1] and no significant interaction effect [*F* (6, 588) = 0.8, *p* = 0.5]. Results are presented in Table 1.

**Table 1:** **Comparison of TCI-140 scores for bipolar patients and comparison subjects**

TCI scale	Bipolar	Controls
	***N*** = 50	***N*** = 50
Novelty Seeking	53.66 ± 9.1	54.2 ± 9.9
Harm Avoidance	61.48 ± 10.8	56.52 ± 11.0
Reward Dependence	67.14 ± 9.0	65.74 ± 9.3
Persistence	63.2 ± 12.9	63.44 ± 10.3
Self-directedness	71.02 ± 12.7	71.02 ± 9.8
Cooperativeness	76.34 ± 9.1	74.42 ± 9.1
Self-transcendence	41.78 ± 11.3	39.98 ± 9.8

Temperament and Character Inventory summed scale scores (mean ± SD) for euthymic patients with bipolar I disorder and for matched healthy comparison subjects. No overall significant differences were found.

### Behavioral addictions

**Figure 1:**
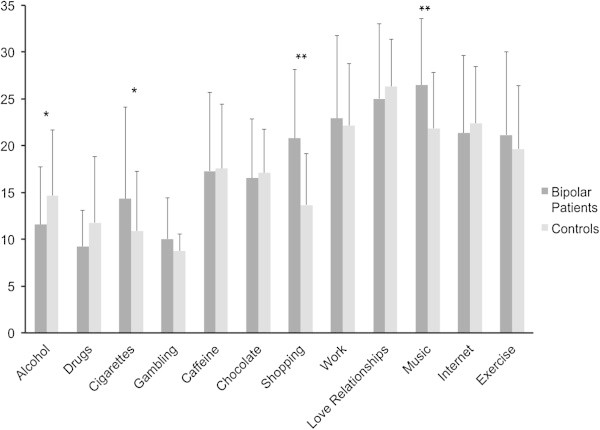
**Comparison of behavioral addiction scores between groups.**

Repeated measures ANOVA with BA domains as within-subject factors showed a significant group X domain interaction [*F* (11,1078) = 6.0, *p* < 0.0001]. Post-hoc (LSD test) comparisons revealed significant differences (*p* < 0.05) on several individual BAs, with BP patients showing higher scores on two benign BAs (music and shopping) and higher scores on smoking. Control group participants showed higher scores on two harmful BAs (drugs (*p* = 0.06) and alcohol (*p* < 0.05)). Results are presented in Figure 1.

### Relationship of TCI and BAs

In order to explore the relationship between temperament/character factors and behavioral addictions, correlations (Pearson’s *r*) were computed between the seven TCI factors and behavioral addictions (grouped as harmful or benign). Results are presented in Table 2. In both patients and controls, novelty seeking was positively correlated with harmful addictions (*p* < 0.03), whereas cooperativeness was negatively correlated with harmful addictions (*p* < 0.05). Self-transcendence was positively correlated with both harmful and benign addictions in controls (*p* = 0.05), but with only benign addictions in patients (*p* < 0.03).

**Table 2:** **Correlations between TCI-140 and Behavioral Addiction Scale scores for bipolar patients and comparison subjects**

TCI scale	Harmful addictions		Benign addictions	
	Patients	Controls	Patients	Controls
Novelty Seeking	0.33*	0.32*	0.22	0.20
Harm Avoidance	−0.14	−0.07	−0.21	0.12
Reward Dependence	−0.09	0.16	0.26	0.01
Persistence	0.11	0.13	0.32*	0.12
Self-directedness	−0.19	−0.05	0.04	−0.26
Cooperativeness	−0.42**	−0.28*	0.02	−0.26
Self-transcendence	0.21	0.29*	0.31*	0.29*

Pearson’s *r* correlations between Temperament and Character Inventory-140 and behavioral addiction scores for euthymic bipolar (*N* = 50) and healthy comparison (*N* = 50) subjects. *p < 0.05; **p < 0.001.

## Discussion

The hypersensitive behavioral approach system model of bipolar disorder would predict higher levels of various addictions in bipolar patients as compared to controls, and in this study, we tested this hypothesis by looking at behavioral addictions in addition to the more commonly studied substance addictions. Euthymic bipolar patients did in fact show significantly elevated levels of two benign behavioral addictions (shopping and music) as well as smoking. Bipolar patients’ levels of drug- and alcohol-related addiction were, surprisingly, lower than those reported by healthy controls. We discuss possible explanations for this below.

The finding of higher tobacco use among BP patients as compared to healthy controls is consistent with other reports (Diaz et al. 2009; Leonard et al. 2001; Itkin et al. 2001). High rates of substance abuse are also often reported among bipolar patients (Lagerberg et al. 2010; Strakowski and DelBello 2000; Swann 2010). We suspect that our finding of lower levels of substance abuse (i.e., illicit drugs and alcohol) among the bipolar subjects may be related to the fact that the bipolar subjects in this study were stable and euthymic and, as a group, have received considerable psychoeducational exposure and are relatively adherent to medication recommendations, as indicated by frequent tests of medication blood levels (see Osher et al. 2010). It also became clear during the testing sessions that many of the bipolar patients had (ab)used drugs and/or alcohol in the past but had modified these behaviors since beginning treatment in the clinic.

This study also explored the relationships between behavioral addictions and personality factors (temperament and character as measured by the TCI-R). In both BP patients and controls, novelty seeking was positively correlated to harmful addictions. This finding is consistent with findings in non-clinical samples regarding consumption of both multiple substances, including illicit drugs and alchohol (Chakroun et al. 2004), and cigarettes (Dinn et al. 2004). The character trait of cooperativeness was found to be negatively related to harmful addictions in both groups. Overall, these findings suggest that temperament and character traits may play roles both as risk factors and as protective factors regarding BAs in euthymic bipolar patients as well as in healthy controls.

One limitation of this study is that the BP patient group consisted mostly of residents of southern Israel, while the control group was recruited mostly from the Sharon area, Israel. Although the distance between the regions is not great, there may be some social or socio-economic differences between the two groups.

## Conclusions

The hypersensitive BAS model of bipolar disorder provides a possible framework for the integration of biological, behavioral, and neurological aspects of this devastating illness. Our findings are largely consistent with this model and contribute to a growing literature, suggesting the importance of continued elucidation of the role of a hypersensitive BAS in the pathogenesis of bipolar affective disorder.

## References

[CR1] Alloy LB, Abramson LY (2010). The role of the behavioral approach system (BAS) in bipolar spectrum disorders. Current Directions in Psychological Science.

[CR2] Alloy LB, Abramson LY, Walshaw PD, Cogswell A, Grandin LD, Hughes ME, Iacoviello BM, Whitehouse WG, Urosevic S, Nusslock R, Hogan ME (2008). Behavioral approach system and behavioral inhibition system sensitivities: prospective prediction of bipolar mood episodes. Bipolar Disorders.

[CR3] Alloy LB, Bender RE, Wagner CA, Whitehouse WG, Abramson LY, Hogan ME, Sylyia LG, Harmon-Jones E (2009). Bipolar spectrum – substance use co-occurrence: behavioral approach system (BAS) sensitivity and impulsiveness as shared personality vulnerabilities. Journal of Personality and Social Psychology.

[CR4] Alloy LB, Abramson LY, Urosevic S, Bender RE, Wagner CA (2009). Longitudinal predictors of bipolar spectrum disorders: a behavioral approach system (BAS) perspective. Clinical Psychology: Science and Practice.

[CR5] Alloy LB, Abramson LY, Walshaw PD, Gerstein RK, Keyser JD, Whitehouse WG, Urosevic S, Nusslock R, Hogan ME, Harmon-Jones E (2009). Behavioral approach system (BAS)-relevant cognitive styles and bipolar spectrum disorders: concurrent and prospective associations. J Abnorm Psychol.

[CR6] Alloy LB, Bender RE, Whitehouse WG, Wagner CA, Liu RT, Grant DA, Jager-Hyman S, Molz A, Choi JY, Harmon-Jones E, Abramson LY (2012). High behavioral approach system (BAS) sensitivity, reward responsiveness, and goal-striving predict first onset of bipolar spectrum disorders: a prospective behavioral high-risk design. J Abnorm Psychol.

[CR7] Berns GS, McClure SM, Pagnoni G, Montague PR (2001). Predictability modulates human brain response to reward. The Journal of Neuroscience.

[CR8] Brown RIF, Eadington WR, Cornelius JA (1993). Some contributions of the study of gambling to other addictions. Gambling behavior and problem gambling.

[CR9] Carver C, White T (1994). Behavioral inhibition, behavioral activation, and affective response to impending reward and punishment: the BIS/BAS scales. Journal of Personality and Social Psychology.

[CR10] Chakroun N, Doron J, Swendsen J (2004). Substance use, affective problems and personality traits: test of two association models. Encephale.

[CR11] Cloninger CR (1999). The Temperament and Character Inventory—Revised.

[CR12] Cloninger CR, Svrakic DM (1997). Integrative psychobiological approach to psychiatric assessment and treatment. Psychiatry.

[CR13] Cloninger CR, Zohar AH (2011). Personality and the perception of health and happiness. Journal of Affective Disorders.

[CR14] Cloninger CR, Svrakic DM, Przybeck TR (1993). A psychobiological model of temperament and character. Archive of General Psychiatry.

[CR15] Depue RA, Collins PF (1999). Neurobiology of the structure of personality: dopamine, facilitation of incentive motivation, and extraversion. Behavioral and Brain Sciences.

[CR16] Depue RA, Iacono WG (1989). Neurobehavioral aspects of affective disorders. Annual Review of Psychology.

[CR17] Di Nicola M, Tedeschi D, Mazza M, Martinotti G, Harnic D, Catalano V, Bruschi A, Pozzi G, Bria P, Janiri L (2010). Behavioral addictions in bipolar disorder patients: Role of impulsivity and personality dimensions. Journal of Affective Disorders.

[CR18] Diaz FJ, James D, Botts S, Maw L, Susce MT, De Leon J (2009). Tobacco smoking behaviors in bipolar disorder: A comparison of the general population, schizophrenia and major depression. Bipolar Disorders.

[CR19] Dinn WM, Aycicegi A, Harris CL (2004). Cigarette smoking in a student sample: neurocognitive and clinical correlates. Addictive Behaviors.

[CR20] Franken IHA, Muris P (2005). BIS/BAS personality characteristics and college students’ substance use. Personality and Individual Differences.

[CR21] Franken IHA, Muris P, Georgieva I (2006). Gray’s model of personality and addiction. Addictive behaviors.

[CR22] Grant JE, Brewer JA, Potenza MN (2006). The neurobiology of substance and behavioral addictions. CNS Spectr.

[CR23] Grant JE, Potenza MN, Weinstein A, Gorelick DA (2010). Introduction to behavioral addictions. The American Journal of Drug and Alcohol Abuse.

[CR24] Gray JA, Royce JR (1973). Causal theories of personality and how to test them. Multivariate analysis and psychological theory (pp.409–463).

[CR25] Gray JA, McNaughton N (2004). The neuropsychology of anxiety: an enquiry into the functions of the septo-hippocampal system (second ed).

[CR26] Itkin O, Nemets B, Einat H (2001). Smoking habits in bipolar and schizophrenic outpatients in southern Israel. Journal of Clinical Psychiatry.

[CR27] Lagerberg TV, Andreassen OA, Ringen PA, Berg AO, Larsson S, Agartz I, Sundet K, Melle I (2010). Excessive substance use in bipolar disorder is associated with impaired functioning rather than clinical characteristics, a descriptive study. BMC Psychiatry.

[CR28] Leonard S, Adler LE, Benhammou K, Berger R, Breese CR, Drebing C, Gault J, Lee MJ, Logel J, Olincy A, Ross RG, Stevens K, Sullivan B, Vianzon R, Virnich DE, Waldo M, Walton K, Freedman R (2001). Smoking and mental illness. Pharmacology, Biochemistry, Behavior.

[CR29] Mardaga S, Hansenne M (2007). Relationships between Cloninger’s biosocial model of personality and the behavioral inhibition/approach systems (BIS/BAS). Personality and Individual Differences.

[CR30] Merikangas KR, Akiskal HS, Angst J, Greenberg PE, Hirschfeld RM, Petukhova M, Kessler RC (2007). Lifetime and 12-month prevalence of bipolar spectrum disorder in the national comorbidity survey replication. Arch Gen Psychiatry.

[CR31] Meyer B, Johnson SL, Winters R (2001). Responsiveness to threat and incentive in bipolar disorder: relations of the BIS/BAS scales with symptoms. Journal of Psychopathology and Behavioral Assessment.

[CR32] Meyer B, Rahman R, Shepherd R (2007). Hypomanic personality features and addictive tendencies. Personality and Individual Differences.

[CR33] Osher Y, Bersudsky Y, Belmaker RH (2010). The new lithium clinic. Neuropsychobiology.

[CR34] Quello SB, Brady KT, Sonne SC (2005). Mood disorders and substance use disorder: a complex comorbidity. Science and Practice Perspective.

[CR35] Salavert J, Caseras X, Torrubia R, Furest S, Arranz B, Dueñas R, San L (2007). The functioning of the behavioral activation and inhibition systems in bipolar I euthymic patients and its influence in subsequent episodes over an eighteen-month period. Personality and Individual Differences.

[CR36] Strakowski MS, DelBello MP (2000). The co-occurrence of bipolar and substance use disorders. Clinical Psychology Review.

[CR37] Swann AC (2010). The strong relationship between bipolar and substance-use disorder. Annals of the New York Academy of Sciences.

[CR38] ten Have M, Vollebergh W, Bijl R, Nolen WA (2002). Bipolar disorder in the general population in the Netherlands (prevalence, consequences and care utilization): results from the Netherlands Mental Health Survey and Incidence Study (NEMESIS). Journal of Affective Disorders.

[CR39] Urosevic S, Abramson LY, Harmon-Jones E, Alloy LB (2008). Dysregulation of the behavioral approach system (BAS) in bipolar spectrum disorders: review of the theory and evidence. Clinical Psychology Review.

[CR40] Zohar AH, Cloninger CR (2011). The psychometric properties of the TCI-140 in Hebrew. European Journal of Psychological Assessment.

